# The Bristol Self Harm Register (BSHR) dataset: Linked self-harm register records of the children in the Avon Longitudinal Study of Parents and Children (ALSPAC)

**DOI:** 10.12688/wellcomeopenres.17724.2

**Published:** 2023-02-01

**Authors:** Mark Mummé, Theresa Redaniel, Andy Boyd, Joni Jackson, Becky Mars, John Macleod

**Affiliations:** 1 Population Health Sciences, University of Bristol, Bristol, BS8 1TH, UK

**Keywords:** Self-harm, mental health, health records, register, record linkage, longitudinal study, cohort study, ALSPAC

## Abstract

This data note describes the linking of records of the Bristol Self Harm Register with the cohort of the index children of the Avon Longitudinal Study of Parents and Children (ALSPAC – also known as ‘Children of the 90s’). These records were obtained from the computerised data base maintained by the Bristol Self Harm Register (BSHR). The BSHR is operated out of the two largest NHS trusts in the ALSPAC study catchment area, North Bristol NHS Trust based at Southmead Hospital and the University Hospitals Bristol and Weston NHS Foundation Trust based at Bristol Royal Infirmary (BRI), both in Bristol. The BSHR database was designed to be populated by staff after an encounter with a patient attending with an indication of self-harm. Some of the information in the BSHR database was self-reported by the patient and was unable to be independently verified. Software syntax was written using STATA (StataCorp LLC, version 17) to convert the original files into a single consistent format in a data base which was reviewed for its potential use in future research. The cleaned BSHR records provide a contemporaneous record of a subset of the ALSPAC cohort over a period of the ALSPAC study in an easily accessible format, which is valuable when other sources of data may be missing.

## Introduction

This data note is written to describe the records of the ALSPAC participants who also have had an episode of self-harm recorded in the Bristol Self-Harm Register and is intended as a tool for researchers considering investigating the area of self-harm and using the ALSPAC resources.

The Avon Longitudinal Study of Parents and Children (ALSPAC) is a multigenerational birth cohort that aims to compile a databank containing information on participants’ health and social exposures and subsequent outcomes across their life course and has been previously described many times ^1– 3^. This Data Note describes the sensitive health data provided via record linkage from the ‘Bristol Self-Harm Register’ electronic records. These records were aimed to be compiled during the participants’ attendances at the local hospitals if there was a reported incident of self-harm. The register started operation in 2010 and provided data to ALSPAC from September 2010, when the youngest ALSPAC children were aged 17 years, up to the end of 2018, the most recent available data set at the time the linkage was performed, and eldest the ALSPAC children were aged 27 years.

The BSHR data can be studied together with the ALSPAC participants’ self-reported data from questionnaires, biological samples, clinical notes, and centrally collected administrative data. It can be used for data quality assessment and to inform missing data strategies. It is worth noting that the BSHR system is a composite of self-reported and abstracted data from within the NHS and there are associated quality issues with the data, which are described in this note.

## Materials and methods

### The ALSPAC sample

The Avon Longitudinal Study of Parents and Children (ALSPAC) is a prospective population-based study which is comprised of the original pregnant women and their partners (these are referred to as Generation Zero or simply G0), their index children (who are ‘The Children of the 90s’, also Generation One or G1) and now the next generation who are called the ‘Children Of the Children Of the 90s, or Cocos, or G2 ^1^.

**Figure 1.**
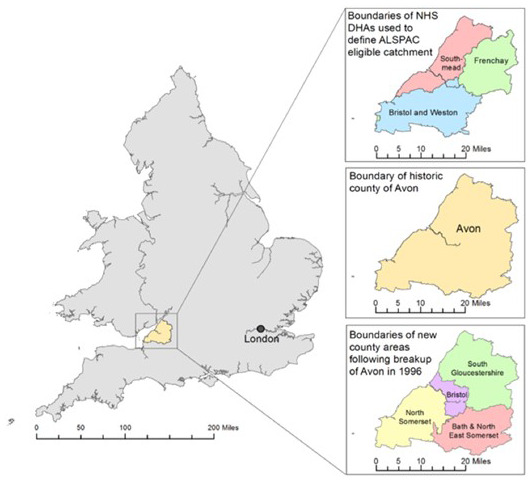
The ALSPAC Eligible Study Area within the UK: illustrating the NHS District Health Authorities (DHAs) used to define: the ALSPAC catchment area; the historical county of Avon; and the four authorities formed following the breakup of Avon. Contains Ordnance Survey, Office of National Statistics and National Records Scotland data © Crown Copyright/database right 2014.

ALSPAC recruited those women who were resident in Avon, UK (former county covering Bristol and the surrounding areas in the Southwest UK) with expected dates of delivery from 1st April 1991 to 31st December 1992 which ultimately resulted in a cohort of 13,988 children who were alive at 1 year of age ^1, 2^. This initial cohort has been supplemented with 913 additional G1 participants who have joined the study since the age of 7, for a total baseline sample of 14,901 G1 participants ^3^. The catchment area is shown in Figure 1 together with the current county boundaries for context.

### The Bristol Self-Harm Register database

The BSHR has been recording detailed information on patients presenting to the hospitals for self-harm since 2010, nearly 20 years after ALSPAC began. University Hospitals Bristol NHS Trust has maintained this database since 2010, primarily in the Emergency Departments of the Bristol Royal Infirmary (BRI) and, since 2013, in Frenchay Hospital and then, following the transfer of acute services, Southmead Hospital of the North Bristol NHS Trust ^4^.

**Figure 2.**
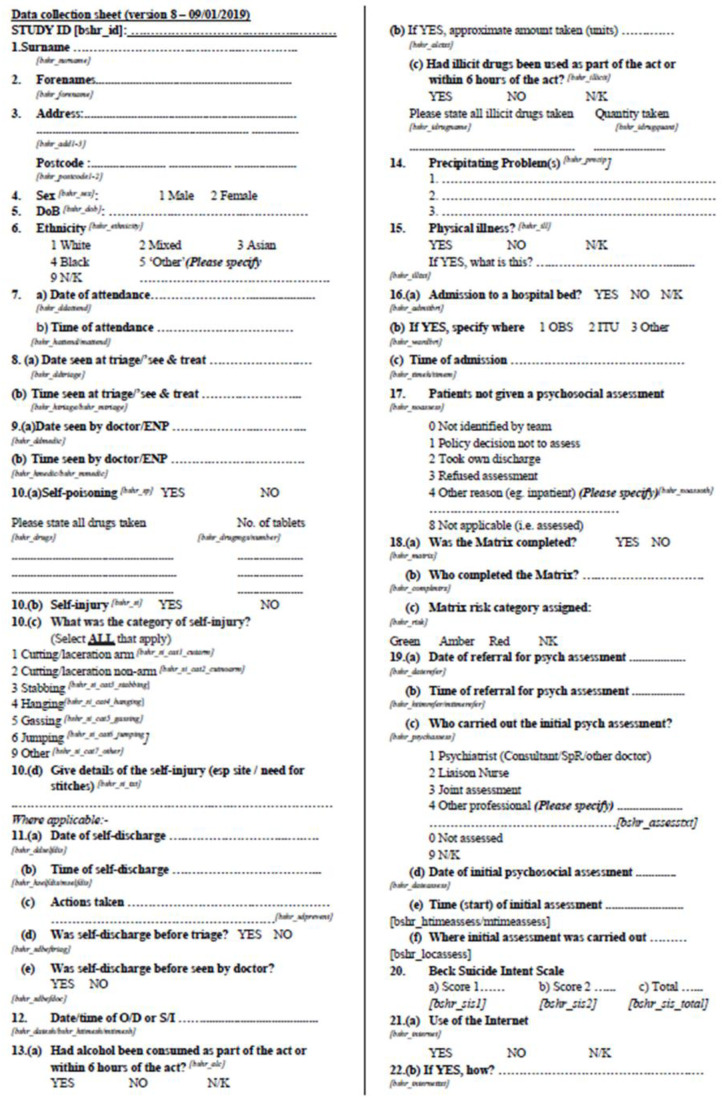
BSHR data collection sheet, version 8.

Emergency department records are searched electronically for potential attendances due to self-harm. Self-harm, for the purpose of the register, is defined as “intentional self-injury or self-poisoning irrespective of motivation or degree of suicidal intent”. The details of the attendances are reviewed to confirm they are related to deliberate self-harm and are then recorded on a database that was developed by the University of Bristol. The information recorded in the database is shown below in Figure 2.

The BSHR is a local dataset which provides a level of detail on the individual’s personal circumstances (e.g.: employment, relationships), precipitating factors leading to the self-harm event, and treatments and referrals which are not found in the national administrative records such as, for example, the NHS-Digital Hospital Episode Statistics (HES) dataset which does, however, include a larger number of individuals. 

There are several sources of data that are collected when compiling information describing these patient attendances for BSHR, including:

i) the liaison psychiatry team’s assessment forms,

ii) the local mental health trust’s patient administration system called “RIO”,

iii) the hospital patient administration system (PAS), and

iv) local Coroners records.

**Figure 3.**
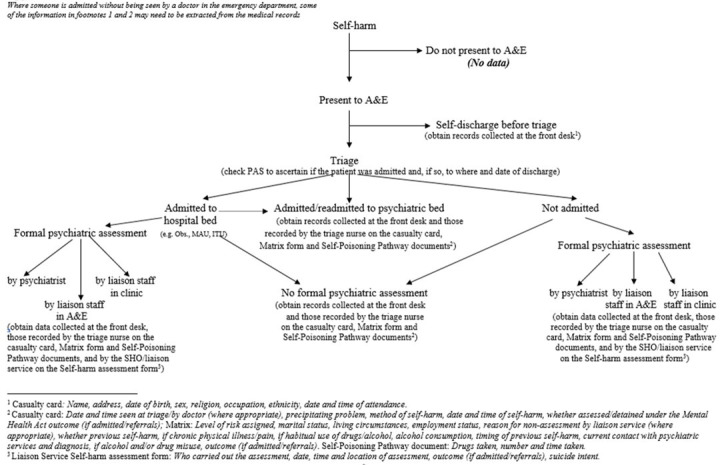
BSHR data collected from each source.

This is shown below in Figure 3.

After all the details of cases identified as self-harm have been recorded onto the database, the data are stored securely on a Trust server. Pseudonymised uploads are sent for analysis to Bristol University.

It is important to note that while the Bristol Self-Harm Register recorded all events of self-harm including those by children as young as 9 years old, the ALSPAC sub-set were all at least 17 years old when the register began in 2010.

### The ALSPAC BSHR dataset

A BSHR data report was extracted for ALSPAC by the BSHR data team. The extract was from two sites, Southmead Hospital of the North Bristol NHS Trust and the Bristol Royal Infirmary of the University Hospitals Bristol and Weston NHS Foundation Trust. These two sites do not cover the entire, original ALSPAC catchment and so the BSHR dataset only includes a subset of this geographic area.

The data selection criteria were based on eligibility criteria for the participant to be enrolled in ALSPAC: which was simply being born of a pregnancy with a due date from 1st April 1991 to 31st December 1992 and within the Avon area. The BSHR facilitated matching to the ALSPAC cohort by selecting a subset of patients from their records with reported dates of birth between 1 ^st^ December 1990 and 31 ^st^ March 1993. Information on the patients, such as NHS numbers and dates of birth, was extracted, and securely provided to the ALSPAC data linkage team, for matching on all individuals present in the BSHR system between these dates of birth regardless of enrolment status in ALSPAC. Some of the entries provided to ALSPAC related to individuals who lived inside of the ALSPAC catchment area and received treatment within the area but were either ineligible or never enrolled in the study, and so these records were deleted.

### Linkage to the ALSPAC database

BSHR staff manually filtered the entries in the register to include only those with a date of birth which fell within the time-period that encompassed all the dates of birth of the original ALSPAC cohort of the index children, G1. The record linkage was achieved through structured querying of the database (i.e., software code that searched a database in a consistent and structured manner) on a case-by-case basis using deterministic approaches using personal identifiers with high levels of discriminatory power (NHS numbers and dates of birth). The match was performed using first the NHS number and then confirming with a date of birth.

### The BSHR research file: denominator

A review of the addresses of the ALSPAC cohort showed that at the age of 17, when the BSHR started in 2010, just over 82% of the participants had an address compatible with attending one of the BSHR sites. This dropped to 70% by age 27 as some of the ALSPAC participants had moved away.

The BSHR research file has been filtered to exclude information on participants who have not enrolled into ALSPAC and those who have subsequently objected to the study’s use of their linked NHS records. Of an original (provided) total of 728 individuals, 464 were dropped as they were not eligible to participate in ALSPAC leaving a possible cohort from BSHR of 264.

**Figure 4.**
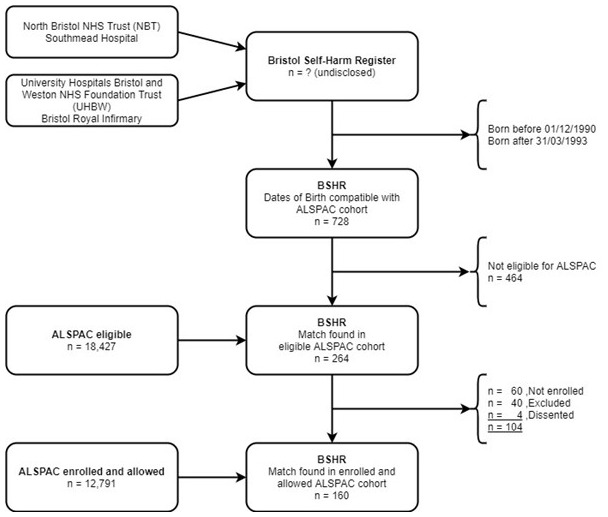
BSHR dataset consort flow diagram.

A further 104 individuals’ records were subsequently removed due to permissions statuses and other factors resulting in a loss of contact over time. These permission statuses include such issues as participants who dissented and withdrew from the study (n=4), study families whose circumstances mean that the study were not able to establish linkage permissions (never enrolled, n=60) and study members who were excluded for a number of reasons (e.g., the G1 child permanently lacks the capacity to consent or the family have requested a break from study contact, n=40). This left a working sample of BSHR records with data on 160 participants ( Figure 4). This represents 1.25% of the 12,791 G1 participants still enrolled in ALSPAC and for whom ALSPAC have permission to access their health records. These numbers are correct at the time of writing. However, the numbers making up the exclusions can be dynamic; for example, as participants change their enrolment or linkage permission status.

### The BSHR research file: data processing

The dataset was provided as an Excel spreadsheet comprising six tables of the raw data as recorded by BSHR. The data was provided as the following tables:

Main –                          the key details of each episode recorded as one line for each episode.

Illicit Drugs –               the details of illicit drugs used as part of the self-harm episode or within 6 hours.

Medication –                details of medication prescribed to the patient at the time of the episode.

Outcome Services –     outcome of the current episode, the patient may possibly be referred to more than one service.

Precipitating Factors – factors reported by the patient as precipitating the current self-harm episode.

Psychiatric Services – psychiatric services referred to after the current episode, the patient may possibly be referred to more than one service.

Software syntax was written using STATA (StataCorp LLC, version 17) to convert the original CSV (comma-separated values) files into a single consistent format. The tables were linked together into a single flat database with each episode recorded as a single row and identifiable data items, such as NHS numbers and dates of birth, were removed.

## The ALSPAC-BSHR dataset

### Patient demographics and backgrounds

The dataset comprised 437 episodes recorded from 160 individuals. There were 59 males (36.9%) who generated 72 records (16.5%), and 101 females (63.1%) who generated the remaining 365 records (83.5%).

The frequency with which participants were recorded in the BSHR, by gender, is listed in Table 1:

**Table 1.** The number of records in the BSHR differentiated by gender.

Number of records per participant	Male	Female
1	48	76
2 +	11	25

There were 43 fully consented participants (26.9%) who generated 274 records (62.7%) and 117 participants (73.1%) whose records were retrieved with the support of section 251 (‘s251’), and these generated 163 records (37.3%). The 43 consented participants were 6 males and 37 females. The 117 participants whose data was provided under section 251 support were 53 males and 64 females.

The ethnicity of the participants recorded within the BSHR was 126 (78.7%) white, 27 (16.9%) either unknown or not recorded. The remaining 7 (4.4%) cannot be further classified without revealing potentially disclosive cell count values of below 5 and so were all recorded as being other than white.

The age in years of participants at the time of a self-harm episode recorded as attending in the BSHR, by gender, is tabulated in Table 2:

**Table 2.** Age in years at the time of the self-harm episode recorded in the BSHR differentiated by gender. [The ‘*’ symbol indicates where small cell counts (<5) have been masked, percentages and totals have also been masked to avoid re-calculation.]

Age (years)	Male		Female		Total
17 *	<5	*-%*	<5	*-%*	<5
18	5	*28%*	13	*72%*	18
19 *	<5	*-%*	33	*-%*	33–38
20	8	*29%*	20	*71%*	28
21	14	*17%*	70	*83%*	84
22	11	*14%*	68	*86%*	79
23 *	<5	*-%*	26	*-%*	26–31
24 *	<5	*-%*	30	*-%*	30–35
25	8	*14%*	51	*86%*	59
26	9	*19%*	38	*81%*	47
27	6	*33%*	12	*67%*	18
unknown *	<5	*-%*	<5	*-%*	<5
	72	*16%*	365	*84%*	437

Marital status was recorded as single for 407 episodes (93.1%). Marital status was recorded as married in 7 episodes (1.6%) and the rest were separated, divorced, not known, or not recorded.

Co-habitation with a partner was recorded in the affirmative for 36 episodes (8.2%) and in the negative for 372 episodes (85.1%). Cohabitation with a partner was recorded as not known or was not recorded for the remaining 29 episodes.

Patient living arrangements were recorded as living alone in 46 episodes (10.5%) and with family, including a partner, in 182 episodes (41.7%). Living arrangements was recorded as ‘other’ in 187 episodes and as not known or was not recorded for the remaining 22 episodes.

There were 11 episodes across 10 individuals who stated they had experienced domestic violence and 8 episodes across 7 individuals who stated they had not. This was recorded as not known or not recorded for the remaining episodes and individuals.

There were 8 episodes across 7 individuals who stated they were LGBT and 11 episodes across <5 individuals who stated they were not. This was recorded as not known or not recorded for the remaining episodes and individuals.

The employment status of the patients at the time of attending for a self-harm episode is shown below in Table 3:

**Table 3.** Employment status recorded by episode.

Employment Status	Frequency (episodes)	Percentage
Employed (including part-time)	92	*21.0%*
Unemployed (seeking employment)	248	*56.8%*
Full-time student	25	*5.7%*
Sickness benefit	12	*2.5%*
Other	11	*2.8%*
Not known, not recorded, data missing	49	*11.2%*

### BSHR sites

There were 331 episodes recorded at the Bristol Royal Infirmary by 113 ALSPAC participants and 106 episodes recorded at Southmead by 47 participants. Table 4, below, shows how the episodes were distributed through time by calendar year between the two sites, also showing the inconsistency with which data was collected in the Southmead site. The total presentations (episodes) to the BSHR are also shown for contrast, although the number of individuals this relates to is unknown.

**Table 4.** The number of records in the BSHR recorded by year between the two sites for ALSPAC participants and total presentations. [ * no or incomplete data collection for this period, small cell counts (<5) have been masked, and totals have also been masked to avoid re-calculation.].

	ALSPAC participant episodes			Total episodes in BSHR
Year	BRI	SMH	Total	
2010	12	n/a *	12	391
2011	33	n/a *	33	1494
2012	31	n/a *	31	1402
2013	36	19	55	2390
2014	59	17 *	76	1813
2015	44	22 *	66	1993
2016	19	<5 *	19–24	1670
2017	46	43	89	3351
2018	51	<5 *	51–56	1750
	331	106	437	16254

### Waiting times

There were 397 episodes where it was possible to calculate the time from attending the Accident and Emergency (A&E) department to being triaged (9 values were not considered because they were either negative or over one hundred hours), and it was observed all 397 considered records were under 3 hours. The mean waiting time for triage was 0.45 hours, it was 0.44 hours for the BRI (304 episodes) and for Southmead (93 episodes) was 0.48 hours. The mean for both males (64 episodes) and females (333 episodes) were 0.45 hours.

There were 351 episodes where it was possible to calculate the time from being triaged to being seen by medical personnel (16 values were not considered because they were either negative or over ten hours), and it was observed all considered records were under 7 hours. The mean waiting time after triage was 1.68 hours, it was 1.52 hours for the BRI (269 episodes) and for Southmead (82 episodes) was 2.04 hours. The mean for males (56 episodes) was 1.43 hours and for females (295 episodes) was 1.68 hours.

There were 69 episodes where it was possible to calculate the time from being seen by medical personnel to being referred for a psychiatric assessment (7 values were not considered because they were either negative or over one hundred hours). For all considered records, the mean waiting time for a psychiatric assessment referral was 4.06 hours, it was 3.53 hours for the BRI (62 episodes) and for Southmead (7 episodes) was 8.73 hours. The mean for males (11 episodes) was 3.47 hours and for females (58 episodes) was 4.17 hours.

There were 56 episodes where it was possible to calculate the time from being referred for a psychiatric assessment to having an initial psychiatric assessment (1 value was not considered because it was either negative or over one hundred hours). For all considered records, the mean waiting time for a psychiatric assessment referral was 7.55 hours, it was 7.51 hours for the BRI (53 episodes) for SMH (3 episodes) was 8.14 hours. The mean for males (11 episodes) was 6.89 hours and for females (45 episodes) was 7.71 hours.

There were 254 episodes where it was possible to calculate the time from first attending A&E to being discharged from the service (3 values were not considered because they were either negative or over one hundred hours). For all considered records, the mean attendance time at the A&E was 11.77 hours, it was 10.30 hours for the BRI (186 episodes) for SMH (3 episodes) was 15.82 hours. The mean for males (33 episodes) was 10.17 hours and for females (45 episodes) was 12.01 hours.

There were 76 episodes were the patient self-discharged, 10 of which were before triage and an additional 32 were before being seen by a doctor.

The time periods between these key stages are summarised below in Table 5:

**Table 5.** Average time-period (hours) between key stages of treatment.

	Average	BRI	SMH	Male	Female
time from attending the A&E to being triaged	0.45	0.44	0.48	0.45	0.45
time from being triaged to being seen by medical personnel	1.68	1.52	2.04	1.43	1.68
time from being seen by medical personnel to being referred for a psychiatric assessment	4.06	3.53	8.73	3.47	4.17
time from being referred for a psychiatric assessment to having an initial psychiatric assessment	7.55	7.51	8.14	6.89	7.71
the time from first attending A&E to being discharged from the service	11.77	10.30	15.82	10.17	12.01

### Method of self-harm

There were 135 individuals, 47 males (34.8%) and 88 (65.2%) females, who together generated 298 (68.2%) episodes recorded in a data field specifically as self-poisoning. There were 4 records with no data on whether they were self-poisoning or not.

There were 34 individuals, 16 males (47.1%) and 18 (52.9%) females, who together generated 185 (42.3%) episodes recorded in a data field specifically as self-injury. There were 6 records with no data on whether they were self-injury or not.

There were 48 episodes from 11 individuals recorded in both the self-poisoning and the self-injury data fields.

The self-injury episodes were broken down into 7 categories, as shown in Table 6a

**Table 6a.** Episode count and participant count, by gender, of self-injury by category.

Categories of Self-Injury	Episodes	Individuals
Cutting/laceration of arm	111	17
Cutting/laceration of non-arm	21	10
Stabbing/Hanging/Gas/Jumping	9	8
Other	13	10

It is important to note that the free text field contained a lot more information than the specific category field. The text was carefully reviewed, and the individual categories were updated to reflect the contents of the self-injury free text field. Not all records had free text associated, and so no categorical data was deleted but categorical data was added when the text described the appropriate self-harm event. The reviewed and updated table of self-injury episodes separated into the same seven categories is shown in Table 6b:

**Table 6b.** Episode count and participant count, by gender, of self-injury by category after review of the free text data.

Categories of Self-Injury	Episodes	Individuals
Cutting/laceration of arm	148	32
Cutting/laceration of non-arm	32	14
Stabbing/Hanging/Gas/Jumping	19	13
Other	21	14

### Precipitating factors

The internet was recorded as being used as part of the episode on 14 occasions (3.2%), however it was not recorded, or recorded as unknown, in a further 320 (73.2%) episodes. There were 10 episodes where internet use was recorded (3 as ‘yes’, 7 as ‘no’) however, in apparent contradiction, it was also recorded that no assessment was performed for these episodes.

Precipitating factors were provided as free text only. These were categorised and re-coded. Many episodes recorded more than one precipitating factor. The categories are shown below in Table 7:

**Table 7.** Frequency of more common precipitating factors of self-harm episodes, as categorised from free-text within the BSHR dataset.

Category of precipitating factor	Episodes
difficulty coping	100
low mood	80
frequent attender	79
suicidal	61
alcohol	49
coping mechanism	48
argument	43
relationship issues	31
BPD (borderline personality disorder)	25
not known	20
stress - other	20
relationship problems	19
voices	18
stress - family	17
drug misuse	16
financial	14
death in family	14
stress at work	13
sleeping	12
psychiatric treatment	12
unemployment issues	10
anger	8
frustrated or fed up	7
intrusive thoughts	7
physical health	6
criminal	6
victim of violence	6
housing	5
relapse mental health	5
anxiety	5
miscarriage	5
*Other with <5 counts: abortion, bereavement* *(other), BOD, dissociation, domestic violence,* *EUPD, expression of emotion, hallucinations,* *hopelessness, isolated, lgbtq+, lost custody of child, * *other, section 136, seeking help,*	28
	789

### Pharmacological factors

Drug and/or alcohol misuse was recorded, separately in specific data fields, as a factor in 223 episodes (28 male and 195 female) and not a factor in 174 episodes (33 male, 141 female). This was recorded as not known or not recorded for the remaining 40 episodes.

Alcohol was consumed as part of the act, or within 6 hours of the act, in 201 episodes (46.0%), and it was not recorded, or recorded as unknown, in a further 50 (11.4%) episodes. Illicit drugs were recorded as consumed as part of the act, or within 6 hours of the act, in 20 episodes (4.6%), and it was not recorded, or recorded as unknown, in a further 56 (12.8%) episodes. Details of illicit drugs taken were recorded in 20 episodes – cannabis x 8, cocaine x 10, and 2 as unknown.

There were 712 records of medication across the 298 episodes with medication details recorded. There were 111 episodes involving 2 or more medications, 44 with 3 or more, 14 with 4 or more and 5 with 5 or more. The medication was recorded as free text, not coded in any way, and so needed cleaning for spelling errors. Medication details included some unknowns and some substances that are not medications but may be used to self-poison (such as bleach or petrol). The most common medications are recorded below in Table 8.

**Table 8.** Frequency of entries recorded as medication taken.

Medication/category	Frequency
Paracetamol	191
Ibuprofen	64
Co-codamol	43
Codeine	26
Zopiclone	25
Citalopram	24
Tramadol	24
Pain Relief	24
Unknown	21
Diazepam	19
Sertraline	19
Sleeping Aid	16
Mirtazapine	16
Cough And Cold	15
Methylphenidate	14
Quetiapine	13
Allergy	11
Warfarin	10

### Assessment and treatment

**Figure 5.**
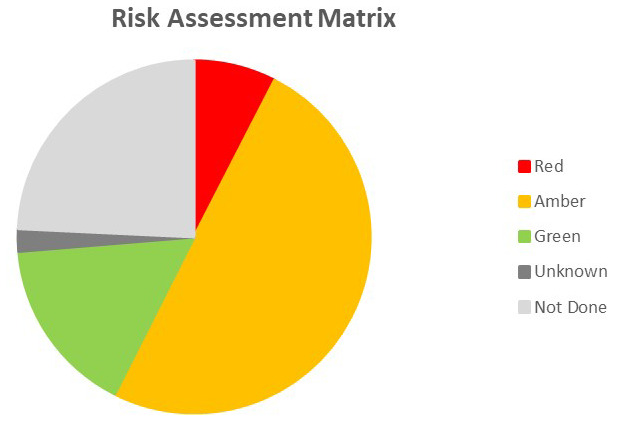
Frequency of episodes with red, amber or green risk assessment matrix score.

A risk assessment matrix was completed as part of 331 episodes (75.7%) as shown in Figure 5 below, and 71 of these were recorded as a green or low, risk, 218 were an amber or medium risk and 33 were a red or high risk. There were 9 matrix assessments where the finding was not recorded or recorded as unknown.

There were 214 (49.0%) episodes which had a psychological assessment, there were 9 episodes where this was not recorded or recorded as unknown. Of these 214 assessments, 145 (67.8%) were done by a liaison nurse and 56 (26.2%) were done by a psychiatrist.

Conversely, there were 223 episodes (51.0%) which had no assessment. The reasons for not assessing these episodes are indicated in Table 9:

**Table 9.** Frequency of reasons for non-assessment of an episode [small cell counts (<5) have been masked].

Reason for non-assessment of episode	Episodes
Not identified by team	50
Policy decision not to assess	60
Took own discharge	64
Refused assessment	16
Other reason	25
N/A (i.e., assessed)	<5
Not recorded, data missing	<5

It is important to note there are 8 episodes with contradictory information between an assessment being done and a reason for non-assessment.

**Figure 6.**
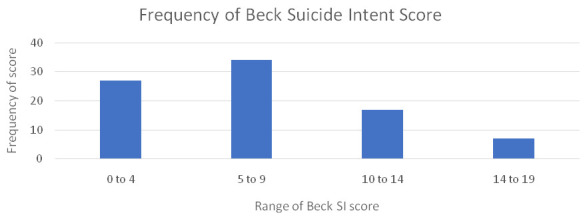
Frequency of episodes with a range of Beck Suicide Intent score.

There were 85 episodes where a Beck Suicide Intent score was calculated. There were 64 episodes with a score up to 10 (the range 1 to 10 is considered a normal range of ups and downs ^2^) and 21 episodes with a score between 10 and 20 (a score of 11 to 16 is categorised as mild mood disturbance and 17 to 20 is borderline clinical depression). This is shown below in Figure 6.

Patients were recorded as being admitted to hospital in 263 episodes (60.2%), 229 were for observation only, just over 30 were admitted to a general ward and <5 were admitted to an intensive care unit.

It was recorded there was a care plan in place for the patient in 140 (32.0%) episodes and there was no care plan in 276 (63.2%). It was recorded as not known or not recorded for the remaining 21 episodes.

### Referrals and Outcomes

The referrals and outcomes were recorded for 433 episodes. There were 55 episodes with more than 1 outcome recorded. There were 19 possible categories provided and the frequency of each outcome is listed in Table 10. There is no further follow-up detail within the register about the accuracy of the outcomes or the duration of, attendance at or success of the referrals; only that the referral was made.

**Table 10.** Frequency of recorded episode outcome [small cell counts (<5) have been masked].

Referral or Outcome	Frequency
Another Community Team	100
Discharged Home/GP care (only)	117
Took Own Discharge	81
Self-Harm Clinic	20
Other Services	94
Bristol Intensive Team	35
Alcohol Misuse Services	<5
Custody (Police/Prison)	22
Died	<5
Samaritans	<5
Crisis House	<5
Psychiatric Inpatient	5
Alcohol Nurse Service	5
Drug Misuse Services	<5
Social Services	<5
IAPT (Improving Access to Psychological Therapies) /LIFT (Least Intervention First Time) LIFT counselling	6
Unspecified	9
CAMHS (Children and Adolescent Mental Health Services)	<5
Domestic Violence Service	<5

It is important to note a key concern on the accuracy of the register is highlighted by the fact that every individual recorded as having died is also recorded as having at least one subsequent episode in the register.

### Physical and mental health

Physical illness was recorded as a factor concurrent with the act in 193 episodes (44.2%), and it was not recorded, or recorded as unknown, in a further 29 (6.6%) episodes. Physical illness was recorded as a factor for 51 (31.9%) of individuals (19 males, 32 females).

The patient affirmed they already had a psychiatric diagnosis in 318 (72.8%) episodes and stated they had not on 68 occasions (15.6%). This was recorded as not known or not recorded for the remaining 51 episodes. Of the 318 episodes when the patient had affirmed that they already had a psychiatric diagnosis, 33 were male and 285 were female.

The diagnoses were recorded by episode, it was observed that more than one diagnosis could be recorded during an episode and that individuals could present with different diagnoses over different episodes. The source of the diagnoses was not provided. The main diagnoses were recorded independently in specific fields and are shown below, in Table 11a. 

**Table 11a.** Psychiatric diagnosis recorded by episode and gender [small cell counts (<5) have been masked].

Psychiatric Diagnosis	Male	Female
Affective	16	39
Personality	<5	209
Psychosis	<5	<5
Organic	<5	<5
Somatoform	<5	<5
Alcohol misuse	<5	12
Drug misuse	<5	<5
Eating disorder	<5	<5
Other	12	128

Some of the diagnoses are described in a free text field and include depression, PTSD, substance misuse, ADHD, panic disorder and autism. It is important to note that the free text field was not always consistent with the specific diagnosis field, for example the field specific to ‘Personality Disorder’ may have a ‘no’ recorded and the associated free text field include ‘BPAD’, ‘BPD’, ‘PD’ or ‘EUPD’ ^3^ or the field specific to ‘Drug Misuse’ may be blank and the text field contain ‘Drug Misuse’. Information on who completed the free text field was not provided. The text was carefully reviewed, and the individual categories were updated to reflect the contents of the free text field. Not all records had free text associated, and so no categorical data was deleted but categorical data was added when the text described the appropriate diagnosis. The reviewed and updated table of diagnosis by episodes separated into the same categories is shown in Table 11b:

**Table 11b.** Psychiatric diagnosis recorded by episode and gender after review of the free text data [small cell counts (<5) have been masked].

Psychiatric Diagnosis	Male	Female
Affective	16	41
Personality	<5	219
Psychosis	<5	<5
Organic	<5	<5
Somatoform	<5	<5
Alcohol misuse	<5	54
Drug misuse	9	28
Eating disorder	<5	68
Other	13	133

The patient affirmed they were already receiving psychiatric care in 277 (63.4%) episodes and stated they were not on 144 occasions (33.0%). This was recorded as not known or not recorded for the remaining 16 episodes.

The patient affirmed they had previous psychiatric care in 315 (72.1%) episodes and stated they had not on 95 occasions (21.7%). This was recorded as not known or not recorded for the remaining 27 episodes.

The patient affirmed they had previously had inpatient psychiatric care within the 12 months prior to their current episode in 37 (8.5%) episodes and they had previously had inpatient psychiatric care more than 12 months prior to their current episode in 26 (6.0%) episodes. They stated they had not had inpatient psychiatric care on 327 occasions (74.8%). This was recorded as not known or not recorded for the remaining 47 episodes.

The patient affirmed they had previously self-harmed in 371 (84.9%) episodes and stated they had not previously self-harmed on 45 occasions (10.3%). This was recorded as not known or not recorded for the remaining 21 episodes.

### Data cross validation

***NHS-Digital.*** NHS-Digital collect national administrative data on all NHS hospital episodes. ALSPAC negotiated an extract of this dataset in 2019. There are 12,336 individuals with at least one entry in ALSPAC’s Hospital Episode Statistics (HES) records from NHS-Digital, which run from 1991 to 2018. Of the 437 BSHR episodes there were 395 which occurred during the period that ALSPAC also had HES records. There were 387 BSHR episodes for which participants had an entry in HES on the same date in both datasets and 8 which did not. All 387 of these episodes had a corresponding entry in Accident and Emergency, 107 also had an entry in Admitted Patient Care as an inpatient and 56 also had an entry in Outpatients; 23 had an entry in all three on the same date.

Table 12 shows how the HES entry recorded the reasons for the 387 A&E episodes:

**Table 12.** Reason for self-harm episodes as recorded in NHS-Digital AE records.

Deliberate self-harm	115
Other than above	257
Other accident or not known	15

The 115 episodes identified in HES as deliberate self-harm, Table 12, involved 58 individuals.

**Figure 7.**
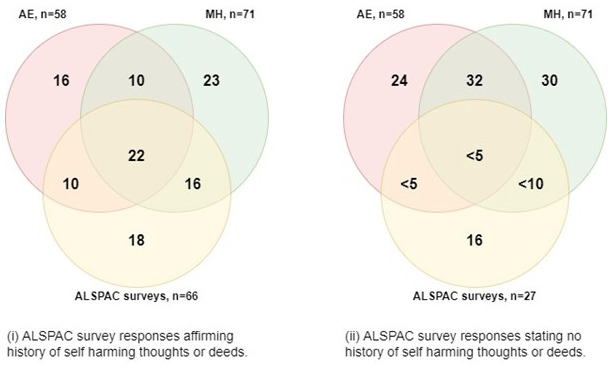
Cross reference of self-harm data of the 160 individuals in the Bristol Self-Harm Register with their NHS-Digital Accident & Emergency (AE) self-harm episodes, their NHS-Digital Mental Health (MH) records, and their ALSPAC self-harm questionnaire question responses [small cell counts (<5) have been masked]. Due to the small number of participants in both the BSHR and the GP records, the primary care records were not included in this graph.

There are 887 individuals from ALSPAC with at least one entry in ALSPAC’s Mental Health (MH) records from NHS-Digital, which run from 2006 to end November 2015. There are 52 individuals from the BSHR who also appear in both the HES and MH datasets during this period. There are a further 19 individuals who are in the MH dataset and appear later in the BSHR, after November 2015 (71 in total). This is shown in Figure 7, below.

**Figure 8.**
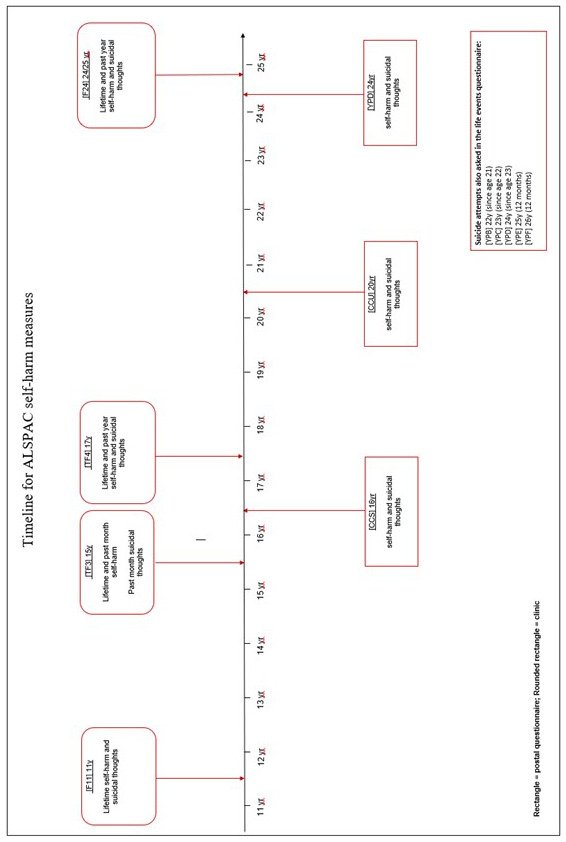
ALSPAC data on Self-Harm, availability by timeline of clinics and questionnaires.

***ALSPAC questionnaires.*** ALSPAC has issued questionnaires to the participants on average more than once every year since it started in 1991. In the ALSPAC survey dataset there are 8,880 individuals who have answered questions about their self-harm history on at least one occasion. The timings of these questions are shown below in Figure 8. Of these, 2,663 (30.0%) affirmed they had engaged in self-harm (regardless of suicidal intent) or had suicidal thoughts, and 6,217 (70.0%) stated they had never engaged in self-harm or had suicidal thoughts.

Of the 160 ALSPAC participants in the BSHR there are 93 (58.1%) who have also responded at least once to an ALSPAC questionnaire about self-harm and 67 (41.9%) about which ALSPAC has no questionnaire data on self-harm. Of the 93 with questionnaire data, 66 (71.0%) stated they had engaged in self-harm or had thoughts of killing themselves and 27 (29%) stated they had not. The records of the 27 who responded that they had never engaged in self-harm or had suicidal thoughts were reviewed further and 24 (88.9%) of these had only responded to ALSPAC questionnaire about self-harm before their first record in the BSHR. There are <5 individuals who stated they had never engaged in self-harm or had suicidal thoughts, but nonetheless had at least one episode recorded in the BSHR.

***Primary Care (GP) data.*** In 2014–16 ALSPAC collected episode data from many GP surgeries in and around the Bristol area, however not all surgeries agreed to share their data, so the primary care dataset is not complete. The data from the GP surgeries was only available up to 2013. There are 11,846 individuals in the ALSPAC primary care dataset with at least one GP record at some point prior to 2013. It is important to note that some of these individuals may have had at least one record in a local GP surgery and other records in other GP surgeries (for example if they changed GP Practice) which either were not approached or did not agree to share their data. There are 105 records indicating a self-harm or suicidal episode involving 59 individuals, from 2003 to 2013. These episodes are divided into 65 which were an overdose event and 40 which were physical self-harm. There are 34 individuals with only one episode in the GP records, 20 for overdose and 14 for self-harm. There are 24 individuals with only overdose events, 8 with both overdose and physical self-harm events and the remainder are physical self-harm only.

During the period from 2010 to 2013, during which the BSHR was also running, there are 53 records of self-harm in the GP dataset, including 40 overdose events, across 26 individuals. During this same period there are 131 records in the BSHR. There are 5 individuals in both the GP and the BSHR datasets. Due to the small numbers, these records were not examined in any detail.

## Ethics

Ethical approval for the study was obtained from the ALSPAC Ethics and Law Committee and the Local Research Ethics Committees. A comprehensive list of research ethics committee approval references is available to download at: http://www.bristol.ac.uk/alspac/researchers/research-ethics/.

The initial approvals for the University of Bristol project were obtained as:

IRAS Number: 54370

NHS REC Reference: 10/H1010/70

## Consent

Permissions for the use of data collected via questionnaires and clinics and record linkage was based on the recommendations of the ALSPAC Ethics and Law Committee and NHS Research Ethics Committee’s at the time. Study participants have the right to withdraw their consent for elements of the study or from the study entirely at any time. Full details of the ALSPAC consent procedures are available on the study website.

## Data Availability

ALSPAC data access is through a system of managed open access. The steps below highlight how to apply for access to the data included in this data note and all other ALSPAC data: i. Please read the ALSPAC access policy ( http://www.bristol.ac.uk/media-library/sites/alspac/documents/researchers/data-access/ALSPAC_Access_Policy.pdf) which describes the process of accessing the data and samples in detail, and outlines the costs associated with doing so. ii. You may also find it useful to browse our fully searchable research proposals database ( https://proposals.epi.bristol.ac.uk/?q=proposalSummaries), which lists all research projects that have been approved since April 2011. iii. Please submit your research proposal ( https://proposals.epi.bristol.ac.uk/) for consideration by the ALSPAC Executive Committee. You will receive a response within 10 working days to advise you whether your proposal has been approved. The availability of our linked participant records is dependent on our ethical approvals and contractual arrangements with the NHS. If you are interested in using these data, then please contact the ALSPAC Data Linkage Team ( alspac-linkage@bristol.ac.uk). This Data Note was conceived by TR who gained the funding needed to support this work. MM drafted the manuscript and conducted the descriptive analyses. TR negotiated access to the BSHR datasets and gained ethical approval for the use of these records. All authors contributed to editing and commenting on the manuscript: **Mark Mumme**, Data Acquisition, Formal Analysis, Investigation, Resources, Software, Writing – Original Draft Preparation **Andy Boyd**, Conceptualization, Project Administration, Writing – Review & Editing **Theresa Redaniel**, Funding Acquisition, Resources, Writing – Review & Editing **Joni Jackson**, Data Acquisition, Writing – Review & Editing **Becky Mars**, Analysis, Writing – Review & Editing **John Macleod**, Funding Acquisition, Supervision, Writing – Review & Editing

## Footnotes

^1^ Details on the cohort profile are available at http://www.bristol.ac.uk/alspac/researchers/cohort-profile/.

^2^ https://www.ismanet.org/doctoryourspirit/pdfs/Beck-Depression-Inventory-BDI.pdf.

^3^ These have been interpreted in order as ‘Borderline Personality Affective Disorder’, ‘Borderline Personality Disorder’, ‘Personality Disorder’ and ‘Emotionally Unstable Personality Disorder’.
