# Supplementary material for: The Bristol Self Harm Register (BSHR) dataset: Linked self-harm register records of the children in the Avon Longitudinal Study of Parents and Children (ALSPAC)
Source: Wellcome Open Res. 2026 Apr 24;7:195. Originally published 2022 Jul 26. [Version 3] doi: 10.12688/wellcomeopenres.17724.3 (PMC13254522; doi:10.12688/wellcomeopenres.17724.3)
Supplement: Peer review history

# Reviewer report: Reviewer response for version 3

**DOI:** 10.21956/wellcomeopenres.29181.r156305

**Published:** 2026-06-22

**Authors:** Georgie Agar

**Affiliations:** 1 School of Life & Health Sciences, Aston University, Birmingham, UK

Thank you for the opportunity to review this data note, which comprehensively describes the linking of ALSPAC data to the Bristol Self-Harm Register. This is an important area of focus for researchers and clinicians and the benefit of hospital data to supplement self-report data on self-harm is clear. The note is mostly clear and well-written, with just a few suggestions for added clarity.

1. There are a few places where correcting minor typos would improve clarity. For example, the last sentence of the second introductory paragraph should read: ‘at the time the linkage was performed the eldest ASPAC children were aged 27 years.’
2. I understand from previous reviews that the Figures are a reflection of the source material. However, Figure 2 is really very difficult to read and seems critical to the reader’s understanding of the data note as a whole. Please could this be typed out/newly created in higher resolution? Unlike Figure 1, there is no reason for it to stay in the current format which is difficult to read. Figure 8 could also be remade to improve clarity.
3. Further details about the Matrix (Figure 2, question 18 and Assessment and treatment section)  would be helpful. What is assessed as part of the Matrix? What do the colour categories relate to, is this risk of future self-harm?
4. Further details about section 251 are needed.
5. Please ensure abbreviations are used consistently throughout. For example the section on waiting times uses both Southmead and SMH which could be confusing.
6. Please specify where the free text injury field comes from (presumably question 10 in Figure 2).
7. Is there a reason why some of the precipitating factors in Table 7 are capitalised and others are not? Is this intended to represent Categories and subcategories within these?
8. What does BOD stand for in the penultimate row of Table 7?
9. Please use an academic citation for the Beck Depression Inventory scoring rather than an online website which could be removed (as in footnote 2).

Are sufficient details of methods and materials provided to allow replication by others?

Partly

Is the rationale for creating the dataset(s) clearly described?

Yes

Are the datasets clearly presented in a useable and accessible format?

Partly

Are the protocols appropriate and is the work technically sound?

Yes

Reviewer Expertise:

Self-injury, paediatrics, longitudinal work

I confirm that I have read this submission and believe that I have an appropriate level of expertise to confirm that it is of an acceptable scientific standard, however I have significant reservations, as outlined above.

# Reviewer report: Reviewer response for version 3

**DOI:** 10.21956/wellcomeopenres.29181.r156299

**Published:** 2026-06-10

**Authors:** Maggie Long

**Affiliations:** 1 Faculty of Arts, Humanities and Social Sciences, Ulster University, Belfast, UK

My only comments relate to presentational issues because the content, methods, and reporting of data look accurate and appropriate to me.

**Introduction**

 Para 2:

 “and eldest the ALSPAC children were aged 27 years.”

 Should this be “the eldest ALSPAC children were”

 Para 3:

 acronym BSHR is used but is not in parentheses in the paragraph above, after the use of the term in full

**Materials and Methods**

 Para 1:

 “which is comprised of the original”

 “Comprised of” is incorrect

 Correct grammar would be, “which comprises the original”

 Consistency in presentation of acronyms below – in full for sub-title, with acronym in parentheses in main paragraph after use of full term:

 “The ALSPAC sample

 The Avon Longitudinal Study of Parents and Children (ALSPAC)”

 “The Bristol Self-Harm Register database

 The BSHR”

**BSHR Sites**

 "Bristol Royal Infirmary by 113 ALSPAC participants and 106 episodes recorded at Southmead": 

 Acronyms BRI and SMH are used inconsistently in section on Waiting Times

 BRI and Southmead are used until: “it was 7.51 hours for the BRI (53 episodes) for SMH (3 episodes)”

 Be consistent and where using acronyms, include in parentheses after first citation of full term.

**Method of Self-harm**

 Is there a table with numbers for self-poisoning? Or just the methods of self-injury?

**Figure 8**

 I had to rotate my laptop to view – can it be included in landscape?

Are sufficient details of methods and materials provided to allow replication by others?

Yes

Is the rationale for creating the dataset(s) clearly described?

Yes

Are the datasets clearly presented in a useable and accessible format?

Yes

Are the protocols appropriate and is the work technically sound?

Yes

Reviewer Expertise:

Self-injury research, social science research on mental health, qualitative research (Grounded Theory).

I confirm that I have read this submission and believe that I have an appropriate level of expertise to confirm that it is of an acceptable scientific standard.

# Reviewer report: Reviewer response for version 2

**DOI:** 10.21956/wellcomeopenres.21002.r54631

**Published:** 2023-03-01

**Authors:** Rosa Alati

**Affiliations:** 1 School of Public Health, Faculty of Health Sciences, Curtin University, Perth, WA, Australia

I have reviewed the changes the authors made and they are satisfactory.

Are sufficient details of methods and materials provided to allow replication by others?

Yes

Is the rationale for creating the dataset(s) clearly described?

Partly

Are the datasets clearly presented in a useable and accessible format?

Yes

Are the protocols appropriate and is the work technically sound?

Yes

Reviewer Expertise:

Longitudinal methodology

I confirm that I have read this submission and believe that I have an appropriate level of expertise to confirm that it is of an acceptable scientific standard.

# Reviewer report: Reviewer response for version 2

**DOI:** 10.21956/wellcomeopenres.21002.r54630

**Published:** 2023-02-22

**Authors:** Jessie R. Baldwin

**Affiliations:** 1 Division of Psychology and Language Sciences, Department of Clinical, Educational and Health Psychology, University College London, London, UK

The authors have made suitable amendments in response to the majority of my points. I have further minor comments regarding the text on internet use as a precipitating factor. 

 First, it’s still unclear why the section on the internet is reported in the text and not in Table 7. If the internet is a precipitating factor recorded in 14 of the episodes, would it not meet criteria for being in the table (which includes factors recorded in 5 or more episodes)? 

 Second, the text is quite difficult to follow: *"The internet was recorded as being used as part of the episode on 14 occasions (3.2%), however it was not recorded, or recorded as unknown, in a further 320 (73.2%) episodes. There were 10 episodes where internet use was recorded (3 as ‘yes’, 7 as ‘no’) however, in apparent contradiction, it was also recorded that no assessment was performed for these episodes."* The first and second sentences appear inconsistent (e.g., 14 episodes versus 3 episodes). And does the latter part mean that we cannot trust the record of it being involved in 10 episodes? I recommend this text be clarified, or preferably the information on the internet be included in the table and not the text for consistency with the other precipitating factors.

Are sufficient details of methods and materials provided to allow replication by others?

Yes

Is the rationale for creating the dataset(s) clearly described?

Partly

Are the datasets clearly presented in a useable and accessible format?

Yes

Are the protocols appropriate and is the work technically sound?

Yes

Reviewer Expertise:

Developmental psychopathology, longitudinal cohort studies, childhood adversity

I confirm that I have read this submission and believe that I have an appropriate level of expertise to confirm that it is of an acceptable scientific standard, however I have significant reservations, as outlined above.

# Reviewer report: Reviewer response for version 1

**DOI:** 10.21956/wellcomeopenres.19612.r52900

**Published:** 2022-12-14

**Authors:** Rosa Alati, Berihun Dachew

**Affiliations:** 1 School of Public Health, Faculty of Health Sciences, Curtin University, Perth, WA, Australia; 2 School of Population Health, Curtin University, Perth, WA, Australia

**Summary: **This data note comprehensively describes the linking of records of the Bristol Self-Harm Register with the cohort of the index children of the ALSPAC, obtained from the computerised database maintained by the Bristol Self-Harm Register (BSHR). Overall, the article is well written and presented with sufficient details of methods and materials. Our comments are as follows:

1. Abstract: The authors stated, ' Software syntax was written using STATA (StataCorp LLC, version 17) to convert the original files into a single consistent format. Could the authors add this information to the method section as well?
2. Page 3: Introduction: The objective and the rationale of this data note is not clearly presented.
3. Page 3: introduction: Any explanations for why Bristol Self-Harm Register (BSHR) stopped providing data to ALSPAC after 2018?
4. Page 3: The ALSPAC Sample: ALSPAC sample description on paragraph 1 requires citation.
5. Page 4: Could the authors describe the methods they used to confirm that the incidents were deliberate self-harm?
6. Page 4: Data collection sheet: The participants' sensitive information, including their name and surname, postcode, sex and DOB, are included in the data collection sheet. I suggest the authors provide information on how this information is handled during data linkage.
7. Page 6: The ALSPAC BSHR dataset: An explanation as to why and how these two sites (SMH and BRI) are selected would be helpful. N.B. The authors stated that these two sites do not cover the entire ALSPAC catchment.
8. Page 6: Page 6: The ALSPAC BSHR dataset: " *The data selection criteria were based on eligibility criteria for the participant to be enrolled in ALSPAC*". Can the authors briefly describe the ALSPC eligibility (other than the DOB, i.e. born before 01/12/1990 and after 31/03/1993?
9. Page 6: Page 6: The ALSPAC BSHR dataset: I suggest rephrasing this sentence for clarity. " *Some of the entries related to individuals who lived inside of the ALSPAC catchment area and received treatment within the area but were either ineligible or never enrolled in the study"*.
10. Page 8: This sentence lacks clarity. " *There were 43 fully consented participants (26.9%) who generated 274 records (62.7%) and 117 participants (73.1%) whose records were retrieved with the support of section 251 ('s251'), and these generated 163 records (37.3%)".*
11. Page 9: Table 4: I suggest putting this ledged in the footnote instead (NOT on the title). “[* no or incomplete data collection for this period.]”
12. Page 10: Participating factors: " *There were 10 episodes where internet use was recorded (3 as 'yes', 7 as 'no') however, it was also recorded that no assessment was performed*". This sentence also lacks clarity.
13. Page 11: Pharmacological factors: are these self-reported? 
14. Page 11: Assessment and treatment "Figure 5 below, and 71 of these were recorded as a green, or low, risk, 218 were an amber, or medium, risk and 33 were a red, or high, risk". Delete comma between the words low, medium and high and risk.
15. Figures: Figure 1 to 4 requires higher resolutions. 
16. Abbreviations: I have noticed many issues in using acronyms.
  1. Always write out the first in-text reference to an acronym (E.g. A$E. IAPT, LIFT and CAMHS).
  2. 'Southmead' may not require an abbreviation. Abbreviations are used to save space and to avoid distracting the reader.
  3. Abbreviations should only be used if the term appears two or more times in the text (e.g. IAPT, LIFT and CAMHS).
  4. Inconsistent use of abbreviations (A&E Vs AE).
17.  Finally, would the authors describe some of the limitations of this data note?

Are sufficient details of methods and materials provided to allow replication by others?

Yes

Is the rationale for creating the dataset(s) clearly described?

Partly

Are the datasets clearly presented in a useable and accessible format?

Yes

Are the protocols appropriate and is the work technically sound?

Yes

Reviewer Expertise:

Longitudinal methodology

We confirm that we have read this submission and believe that we have an appropriate level of expertise to confirm that it is of an acceptable scientific standard, however we have significant reservations, as outlined above.

# Response

**Published:** 2023-02-08

**Authors:** Mark Mumme

**Affiliations:** University of Bristol, UK

1. Abstract: The authors stated, ' Software syntax was written using STATA (StataCorp LLC, version 17) to convert the original files into a single consistent format. Could the authors add this information to the method section as well?

*I have now included a reference to STATA in the method section.*

 2. Page 3: Introduction: The objective and the rationale of this data note is not clearly presented.

*A short paragraph has been added to the introduction to further explain the objective of this data note. Further rationale has been added to the end of the section ‘The Bristol Self-Harm Register database’.*

 3. Page 3: introduction: Any explanations for why Bristol Self-Harm Register (BSHR) stopped providing data to ALSPAC after 2018?

*An explanation has been added that 2018 was the most recent available data set at the time the linkage was performed.*

 4. Page 3: The ALSPAC Sample: ALSPAC sample description on paragraph 1 requires citation.

*References are included to explain the ALSPAC sample/cohort.*

 5. Page 4: Could the authors describe the methods they used to confirm that the incidents were deliberate self-harm?

*Some detail of the process is now included at page 3, the section ‘The Bristol Self-Harm Register database’, describing how the cases were selected for the register. For clarity, this was not done by the author, the author is describing the register after it was constructed.*

 6. Page 4: Data collection sheet: The participants' sensitive information, including their name and surname, postcode, sex and DOB, are included in the data collection sheet. I suggest the authors provide information on how this information is handled during data linkage.

*The linkage process using personal identifiers is described page 4, section ‘Linkage to the ALSPAC database’.*

 7. Page 6: The ALSPAC BSHR dataset: An explanation as to why and how these two sites (SMH and BRI) are selected would be helpful.

*N.B. The authors stated that these two sites do not cover the entire ALSPAC catchment. The sites which have hosted the BSHR are described more fully at page 3, the section ‘The Bristol Self-Harm Register database’.*

 8. Page 6: Page 6: The ALSPAC BSHR dataset: "The data selection criteria were based on eligibility criteria for the participant to be enrolled in ALSPAC". Can the authors briefly describe the ALSPC eligibility (other than the DOB, i.e. born before 01/12/1990 and after 31/03/1993?

*The ALSPAC eligibility is described more fully at page 3, the section ‘The ALSPAC Sample’ and the references cited within. It was not considered necessary to repeat all the details for this section. * 

 9. Page 6: Page 6: The ALSPAC BSHR dataset: I suggest rephrasing this sentence for clarity. "Some of the entries related to individuals who lived inside of the ALSPAC catchment area and received treatment within the area but were either ineligible or never enrolled in the study".

*This sentence has been expanded slightly, to clarify that although these records were provided to ALSPAC they were not relevant to the study, and so were deleted.*

 10. Page 8: This sentence lacks clarity. "There were 43 fully consented participants (26.9%) who generated 274 records (62.7%) and 117 participants (73.1%) whose records were retrieved with the support of section 251 ('s251'), and these generated 163 records (37.3%)".

*This sentence, although dense with numbers, is correct and has not been altered.*

 11. Page 9: Table 4: I suggest putting this ledged in the footnote instead (NOT on the title). “[* no or incomplete data collection for this period.]”

*This has not been moved to a footnote, but is clearly differentiated from the title. * 

 12. Page 10: Participating factors: "There were 10 episodes where internet use was recorded (3 as 'yes', 7 as 'no') however, it was also recorded that no assessment was performed". This sentence also lacks clarity.

*A small section, ‘The Internet’, has been created for clarity, and this wording has been updated.*

 13. Page 11: Pharmacological factors: are these self-reported?

*Although the data was input in a hospital setting, it could not be confirmed whether or not the source of these were self-reported or from clinical samples.*

 14. Page 11: Assessment and treatment "Figure 5 below, and 71 of these were recorded as a green, or low, risk, 218 were an amber, or medium, risk and 33 were a red, or high, risk". Delete comma between the words low, medium and high and risk.

*This sentence has been edited as suggested.*

 15. Figures: Figure 1 to 4 requires higher resolutions.

*The quality of the images are a reflection of the source material.*

 16. Abbreviations: I have noticed many issues in using acronyms.

1. Always write out the first in-text reference to an acronym (E.g. A$E. IAPT, LIFT and CAMHS).
2. 'Southmead' may not require an abbreviation. Abbreviations are used to save space and to avoid distracting the reader.
3. Abbreviations should only be used if the term appears two or more times in the text (e.g. IAPT, LIFT and CAMHS).
4. Inconsistent use of abbreviations (A&E Vs AE).

*The use of acronyms/initialisms has been reviewed and reduced throughout the document. * 

 17. Finally, would the authors describe some of the limitations of this data note?

*The author has attempted to faithfully describe the data (the raw numbers, categories, strengths, weaknesses, inconsistencies) while trying to avoid, as is right with a data note, drawing a conclusion.*

# Reviewer report: Reviewer response for version 1

**DOI:** 10.21956/wellcomeopenres.19612.r51694

**Published:** 2022-08-09

**Authors:** Jessie R. Baldwin

**Affiliations:** 1 Division of Psychology and Language Sciences, Department of Clinical, Educational and Health Psychology, University College London, London, UK

This data note comprehensively describes the linking of medical records on self-harm from the Bristol Self-Harm Register to the ALSPAC dataset. The use of medical records to assess self-harm will be valuable to researchers using ALSPAC, as these are objective records that do not rely on self-report (and thus are not subject to recall bias or shared method variance, when examining other self-reported measures). The authors should be commended for linking these data and providing a detailed description of these measures in this article. Below I list suggestions for improving the data note.

 It would be helpful if the authors could add information on the following questions:

1. Do the authors recommend that researchers wanting to look at records of self-harm in ALSPAC request these data alongside all the other health records (e.g., Hospital Episode Statistics, Mental Health records from NHS Digital, and GP Primary Care data) for a more complete picture?
2. What is the added value of the BSHR over the health records on self-harm already linked to ALSPAC?
3. What was the proportion of eligible ALSPAC participants covered by the relevant hospitals included in the BSHR?
4. What should researchers using these data do to address the fact that only a subset of ALSPAC participants could be included in the BSHR, due to their location (as only 2 hospitals collected these self-harm records)?

Further suggestions:

1. Page 1 (P1), last sentence - could the authors provide the ages of the ALSPAC participants that correspond to these years captured by the BSHR?
2. On a related note, a helpful timeline for ALSPAC questionnaire measures on self-harm is provided. It would be very useful if the authors could edit this to show the age ranges of self-harm measures covered by the different administrative records, including the BSHR.
3. When referring the reader to tables with categories (e.g., self-injury episodes, precipitating factors), it would be helpful to add some text explaining what the most and least common categories were.
4. P8 – ethnicity information: I recommend providing a breakdown of the different ethnic groups rather than saying “other than white” which is othering.
5. Table 2 – it was not clear to me why some cells had missing information on percentage and why some cells in the “Total” column had a range of numbers. Could the authors provide a note explaining this or edit the table?
6. P10 – precipitating factors: it was not clear why the internet was discussed in the text but was not represented in Table 7.
7. P11 – Could the authors explain why the Beck Suicide Intent cut off of 10 was used to dichotomise episodes?
8. P12 – Outcomes: Could the authors add how outcomes were assessed – e.g., what was the length of follow-up, were outcomes those that were recorded in the BSHR records?  
9. As a general point, unless there is a strict word count, it would be helpful to avoid acronyms unless used very frequently (e.g., BSHR), to help readers follow the text more clearly.
10. Finally, could the figures all be provided in higher resolution so readers can view them more clearly?

Are sufficient details of methods and materials provided to allow replication by others?

Yes

Is the rationale for creating the dataset(s) clearly described?

Partly

Are the datasets clearly presented in a useable and accessible format?

Yes

Are the protocols appropriate and is the work technically sound?

Yes

Reviewer Expertise:

Developmental psychopathology, longitudinal cohort studies, childhood adversity

I confirm that I have read this submission and believe that I have an appropriate level of expertise to confirm that it is of an acceptable scientific standard, however I have significant reservations, as outlined above.

# Response

**Published:** 2023-02-08

**Authors:** Mark Mumme

**Affiliations:** University of Bristol, UK

1. Do the authors recommend that researchers wanting to look at records of self-harm in ALSPAC request these data alongside all the other health records (e.g., Hospital Episode Statistics, Mental Health records from NHS Digital, and GP Primary Care data) for a more complete picture?

*A paragraph has been added to the ‘Introduction‘ to address this.*

 2. What is the added value of the BSHR over the health records on self-harm already linked to ALSPAC?

*A paragraph has been added to section ‘The Bristol Self-Harm Register database‘ to address this.*

 3. What was the proportion of eligible ALSPAC participants covered by the relevant hospitals included in the BSHR?

*A paragraph has been added to section ‘The BSHR research file: denominator‘ to address this – “in 2010, just over 82% of the participants had an address compatible with attending one of the BSHR sites. This dropped to 70% by age 27 as some of the ALSPAC participants had moved away”.*

 4. What should researchers using these data do to address the fact that only a subset of ALSPAC participants could be included in the BSHR, due to their location (as only 2 hospitals collected these self-harm records)?

*The author has made the reader aware that missingness is a factor in using this data, but does not make any specific recommendations on how this should be addressed.*

 Further suggestions:

 1. Page 1 (P1), last sentence - could the authors provide the ages of the ALSPAC participants that correspond to these years captured by the BSHR?

*This information has been added. * 

 2. On a related note, a helpful timeline for ALSPAC questionnaire measures on self-harm is provided. It would be very useful if the authors could edit this to show the age ranges of self-harm measures covered by the different administrative records, including the BSHR.

*The author did not think this appropriate because ALSPAC tries to regularly update the administrative data available and dates published in this data note regarding other datasets would quickly become out of date.*

 3. When referring the reader to tables with categories (e.g., self-injury episodes, precipitating factors), it would be helpful to add some text explaining what the most and least common categories were.

*The author does highlight some key numbers or rates from the tables while trying to avoid replicating too many details of the table in the text.*

 4. P8 – ethnicity information: I recommend providing a breakdown of the different ethnic groups rather than saying “other than white” which is othering.

*The ‘other than white’ ethnicities could not be broken down further due to very small numbers and the policy of masking counts less than five, so the sentence has been reworded to explain this.*

 5. Table 2 – it was not clear to me why some cells had missing information on percentage and why some cells in the “Total” column had a range of numbers. Could the authors provide a note explaining this or edit the table?

The following explanation has been added – “The ‘*’ symbol indicates where small cell counts (<5) have been masked, percentages and totals have also been masked to avoid re-calculation.”

 6. P10 – precipitating factors: it was not clear why the internet was discussed in the text but was not represented in Table 7.

*A small section, ‘The Internet’, has been created for clarity, and the wording has been expanded.*

 7. P11 – Could the authors explain why the Beck Suicide Intent cut off of 10 was used to dichotomise episodes?

*The portion relating to the Beck Suicide Intent score has been reworked using the scores in line with the defined categories. The author thanks the reviewer for highlighting this.*

 8. P12 – Outcomes: Could the authors add how outcomes were assessed – e.g., what was the length of follow-up, were outcomes those that were recorded in the BSHR records?

*A sentence has been added – “There is no further follow-up detail within the register about the accuracy of the outcomes or the duration of, attendance at or success of the referrals; only that the referral was made.”*

 9. As a general point, unless there is a strict word count, it would be helpful to avoid acronyms unless used very frequently (e.g., BSHR), to help readers follow the text more clearly.

*The use of acronyms/initialisms has been reviewed and reduced throughout the document.*

 10. Finally, could the figures all be provided in higher resolution so readers can view them more clearly?

*The quality of the images are a reflection of the source material.*
